# Improved Production of 5-Hydroxymethylfurfural in Acidic Deep Eutectic Solvents Using Microwave-Assisted Reactions

**DOI:** 10.3390/ijms23041959

**Published:** 2022-02-10

**Authors:** Eduarda S. Morais, Mara G. Freire, Carmen S. R. Freire, Armando J. D. Silvestre

**Affiliations:** Chemistry Department, Portugal Campus Universitário de Santiago, CICECO-Aveiro Institute of Materials, University of Aveiro, 3810-193 Aveiro, Portugal; morais.eduarda@ua.pt (E.S.M.); maragfreire@ua.pt (M.G.F.); cfreire@ua.pt (C.S.R.F.)

**Keywords:** 5-hydroxymethylfurfural, deep eutectic solvents, catalyst, fructose

## Abstract

Hydroxymethylfurfural (5-HMF) is a key platform chemical, essential for the production of other chemicals, as well as fuels. Despite its importance, the production methods applied so far still lack in sustainability. In this work, acidic deep eutectic solvents (DES), acting both as solvent and catalyst, were studied for the conversion of fructose into 5-HMF using microwave-assisted reactions. These solvents were screened and optimized by varying the hydrogen bond donor (HBD) and hydrogen bond acceptor (HBA). The bio-based solvent γ-valerolactone (GVL) was also applied as additive, leading to a boost in 5-HMF yield. Then, a response surface methodology was applied to further optimize operating conditions, such as reaction time, temperature and wt.% of added GVL. The highest 5-HMF yield attained, after optimization, was 82.4% at 130 °C, in 4 min of reaction time and with the addition of 10 wt.% of GVL. Moreover, a process for 5-HMF recovery and DES reuse was developed through the use of the bio-based solvent 2-methyltetrahydrofuran (2-Me-THF), allowing at least three cycles of 5-HMF production with minimal yield losses, while maintaining the purity of the isolated 5-HMF and the efficacy of the reaction media.

## 1. Introduction

The instability of the hydrocarbons market as well as the rising concerns about global warming and the diminishing of fossil resources has led to a paradigm shift from a fossil to a bio-based economy [1]. Biomass is an abundant and renewable feedstock to be applied in this context. It is mainly composed of carbohydrates, which can be isolated and converted into a plethora of chemicals, materials and fuels. For instance, it is estimated that by 2050, 30% of all chemicals should be derived from biomass [2,3]. However, biomass is a complex feedstock and thus the development of efficient and sustainable methods for its fractionation and for the transformation of the carbohydrate fraction [4,5]. Among the compounds derived from carbohydrates, furans are considered to have a high potential for the production of chemicals, materials and fuels [6]. In fact, furans, such as furfural, 5-hydroxymethylfurfural (5-HMF) and 2,5-furandicarboxylic acid have been considered by the U.S. Department of Energy (DOE) amongst the key chemicals attained from biomass sources [7]. 5-HMF is also known as the “sleeping giant” due to its potential as a platform chemical in the chemical industry [8]. For example, this compound is a versatile intermediate that can be converted into 2,5-furandicarboxylic acid, 2,5-dihydroxymethylfuran and 2,5-bis(hydroxymethyl)tetrahydrofuran, which can be used to replace building blocks in the production of polyesters, and dimethyl-furan that can be used in the liquid transportation of fuel [6,9,10,11]. Additionally, 5-HMF can also be transformed into liquid alkanes through a multitude of reaction pathways, with high potential as fuels [1,10].

5-HMF can be produced by the direct dehydration of hexoses, such as fructose and glucose, or by the hydrolysis and dehydration of disaccharides, trisaccharides, and polysaccharides, either in single or biphasic systems [11]. When the reactions’ starting point is glucose, generally isomerization to fructose takes place since it favors 5-HMF production [12]. Three mechanisms for the production of 5-HMF from sugars have been proposed. The first one, and most commonly used, is the direct formation to 5-HMF by acid-catalyzed dehydration of hexoses, in which three water molecules are consecutively removed from the sugar molecule; the second route is through Maillard reactions in hexoses which is only possible in the presence of amino acids and amines; and the third route is through aldol condensation reactions of smaller C3 molecules [12]. Furthermore, while 5-HMF can be produced directly by the dehydration of hexoses, when disaccharides, such as saccharose or polysaccharides such as starch, inulin or cellulose are applied, hydrolysis into the corresponding C-6 monosaccharides are steps that need to occur before dehydration [8,13]. In literature, these conversions have been carried out in aqueous systems with the use of mineral acids, such as hydrochloric (HCl) [14] sulphuric (H_2_SO_4_) [15] and phosphoric (H_3_PO_4_) [16] acids or organic acids, for instance, formic acid (H_2_CO_2_) [17]. Often, the reactions are performed in organic solvents, including dimethylsulfoxide (DMSO) [13] and dimethylformamide (DMF) [18]. Moreover, heterogeneous metallic catalysts such as aluminum oxide (Al_2_O_3_) [19] zinc oxide (ZrO_2_) [19] chromium chloride (CrCl_2_) [20] among many others, are frequently employed to increase conversion and 5-HMF yield. Eco-friendly and bio-based solvents, such as γ-valerolactone (GVL), have also been tested as substitutes of water or in mixtures with water in dehydration reactions with mineral acids. The use of this polar aprotic solvent allows to decrease the reaction activation energy and increase the overall yield [21]. Nonetheless, and despite the extensive literature on the synthesis of 5-HMF, the selectivity of the process, 5-HMF degradation and its purification are issues that still hamper further industrialization.

Many types of secondary reactions may occur, such as the rehydration of 5-HMF and subsequent conversion to formic and levulinic acids or the production of humins through condensation [22]. A possible way to circumvent these issues is by the use of biphasic systems composed of an aqueous and an organic phase. The use of this strategy allows for the in-situ extraction of the generated 5-HMF, thus preventing degradation, concentrating the product and thermodynamically shifting the reaction towards the product formation [11]. Homogeneous or heterogeneous catalysts are included in the aqueous media while the organic phase can consist of solvents, such as methyl isobutyl ketone (MIBK) [23] dichloromethane [24] and 2-butanol [25]. The use of microwave-assisted reactions is another methodology that can increase the efficiency of 5-HMF production. This methodology is commonly used in organic synthesis and has proven to be very efficient in the dehydration of fructose, highly reducing the reaction time and increasing the selectivity and yields [26]. For example, a fructose yield of 95% was achieved using MW irradiation for 1 min at 200 °C, using only an aqueous solution of 0.01 M HCl [14].

The use of “designer solvents”, specifically ionic liquids (ILs) and deep eutectic solvents (DES) has also gained interest in 5-HMF production [6]. ILs are a class of low melting salts with relevant properties, such as low vapor pressure and good chemical and thermal stability, making them ideal as solvents for the production of 5-HMF [27,28]. They have been extensively applied to this purpose, most often in conjunction with heterogeneous catalysts and/or with biphasic systems [28]. Zhao et al. [5] used 1-butyl-3-methylimidazolium ([C_4_C_1_im]Cl), 1-ethyl-3-methylimidazolium ([C_2_C_1_im]Cl), and 1-octyl-3-methylimidazolium ([C_8_C_1_im]Cl) in combination with metal halides to convert fructose and glucose into 5-HMF. Such systems led to 5-HMF yields ranging from 70% (with the use of CrCl_2_) and 83% (while using RhCl_3_ or PtCl_2_), at 80 °C for 3 h [5]. In a more complex system, -methyl-3-(butyl-4-chlorosulfonyl) imidazolium chlorosulfate ([C_1_C_4_ClIm][SO_3_Cl]) was used in a ternary system composed of acetonitrile and water at room temperature for the dehydration of fructose to 5-HMF. With this system, a yield of 88.7% of 5-HMF in the acetonitrile phase was obtained and the authors claimed that the IL could be easily removed by treatment with alumina [29].

The use of DES in 5-HMF production, on the other hand, is more recent [22]. These solvents, if properly designed, are cheaper to produce and may disclose low toxicity, especially those derived from renewable resources [30]. They were first reported by Abbott et al. [31] as mixtures of solids with lower melting points than each of the individual components. This melting point depression is due to the hydrogen bonding network established amongst components and to the charge delocalization resulting from it [32,33]. DES are composed of at least one hydrogen bond donor (HBD) and one hydrogen bond acceptor (HBA) and can be easily prepared by mixing both components in specific molar ratios. Han and co-workers [34] produced 5-HMF from fructose with 45%, 61%, and 84% selectivity in the presence of [Ch]Cl:malonic acid, [Ch]Cl:oxalic acid, and [Ch]Cl:citric acid monohydrate, respectively, at 80 °C for 1 h. When ethyl acetate was used as an extractant phase, the yields attained with [Ch]Cl:citric acid were boosted up to 85%; however, the 5-HMF extraction was lower than 65% due to its distribution between the two phases [34]. More recently, acidic-based DES coupled with metallic catalysts were applied in a biphasic system with MIBK to produced 5-HMF from different carbohydrate sources [35]. The reactions were carried out at 130 °C for 2 h and the yields attained varied from 63.3% to 54.5%, for glucose and bread waste, respectively [35]. Moreover, [Ch]Cl seems to play an important role in the production of 5-HMF, by stabilizing this compound in the reactive mixture and enhancing its yield [22,35]. Nonetheless, this affinity may also hinder 5-HMF separation from the DES media, and thus new and effective separation methods are required [22].

In this work, several acidic-based DES have been applied, as both solvent and catalyst, for the microwave-assisted production of 5-HMF from fructose. Microwave heating was used to further increase 5-HMF yields and decrease the reaction time. Different DES were screened to determine the most efficient for 5-HMF production by varying both the HBD and HBA. 5-HMF yields were improved by the addition of γ-valerolactone (GVL) to the reaction media, facilitating the manipulation of the system and taking advantage of its positive impact on this type of reaction as referred to above [21]. The process was then optimized regarding temperature, time and the amount of GVL added. Finally, a process for the DES reuse and 5-HMF recovery was developed using the bio-based solvent 2-methyltetrahydrofuran (2-MeTHF). To the best of our knowledge, this is the first attempt to develop a fully integrated process for 5-HMF production allying the use of acidic DES with microwave-assisted reactions while also focusing on product recovery and integrity, as well as solvent re-use.

## 2. Results and Discussion

**Figure 1:**
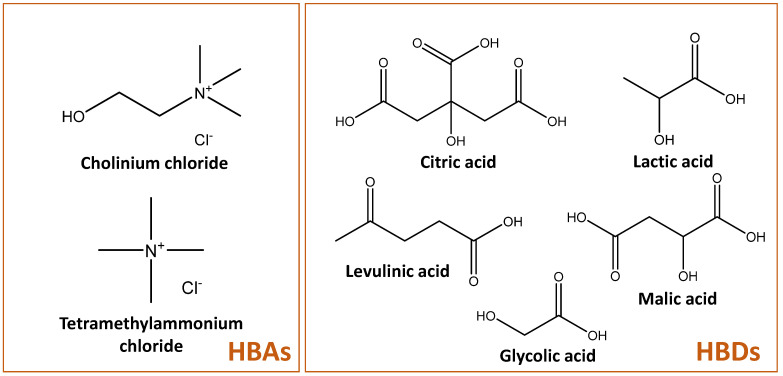
Structure of the HBAs and HBDs discussed in this work.

In this work, fructose was used as a substrate for the production of 5-hydroxymethylfurfural through microwave-assisted reactions. An initial DES screening was carried out through microwave-assisted reactions and a comparison was established between the performance of DES in a combination of several HBDs and two different HBAs (Figure 1). After the selection of the best HBD candidate, the HBA anions were varied to study their effect in the 5-HMF yields. After the selection of the DES that allowed higher yields, a response surface methodology (RSM) approach was used to optimize several parameters, namely reaction time, temperature and the amount of the co-solvent utilized (GVL).

### 2.1. Initial DES Screening

**Figure 2:**
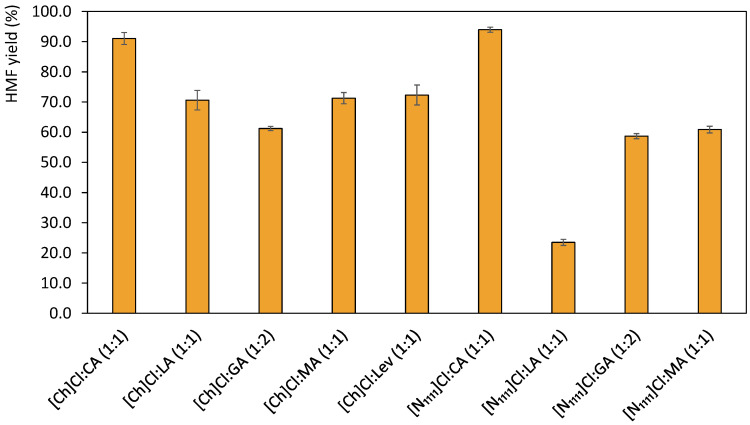
5-HMF yields attained for different DES at 120 °C, 2 min of reaction time with microwave heating and S/L ratio of 0.05.

The DES based on cholinium chloride ([Ch]Cl) and tetramethylammonium chloride ([N_1111_]Cl) as HBAs and citric (CA), lactic (LA), glycolic (GA), malic (MA) and levulinic (Lev) acids as HBDs were prepared in a 1:1 molar ratio, when possible, with the exception of the DES based in glycolic acid since no DES formation was observed for ([N_1111_]Cl:GA) in the molar ratio of 1:1, and thus a 1:2 ratio was used. Moreover, it was not possible to produce a DES with [N_1111_]Cl:Lev in any appropriate molar ratio, being this combination discarded. The prepared DES were then tested for 5-HMF production in the following conditions: 120 °C and 2 min of reaction time, using 150 mg of fructose as substrate and 3 g of DES (solid-liquid, S/L, ratio of 0.05). The obtained results are depicted in Figure 2.

As observed in Figure 1 yields above 50% were attained for all DES combinations, with the exception of [N_1111_]Cl:LA (1:1). Moreover, when comparing the HBAs [N_1111_]Cl and [Ch]Cl, the latter generally promotes higher 5-HMF yields, with the exception of the DES with GA and CA in which very similar 5-HMF yields were obtained. Citric acid was the HBD that resulted in the best yields for both HBAs, namely 91.03% for [Ch]Cl:CA and 93.93% for [N_1111_]Cl:CA. The high yields reached in these cases could be mainly related to the use of citric acid as HBD. This acid has the lowest pKa and is a tricarboxylic acid, thus resulting in a faster promotion of the dehydration reaction [36]. The results attained surpass those obtained by Han and co-workers [9] with [Ch]Cl:CA, in which they reported 75% of yield during 1 h at 80 °C under conventional heating [9]. This difference in yield can be attributed to the use of microwave-assisted reactions, as well as to the DES thermal stability and capacity to absorb microwave radiation.

Through the analysis of the results of this initial screening, citric acid is shown to be the best candidate to be used as the HBD component of the DES. On the other hand, [Ch]Cl and [N_1111_]Cl display very similar results when paired with CA. Nonetheless, the use of [Ch]Cl is preferred since this compound is less expensive than [N_1111_]Cl, contributing to a decrease in the overall cost of the process. Therefore, the cholinium cation was selected for the next steps of the process optimization.

**Figure 3:**
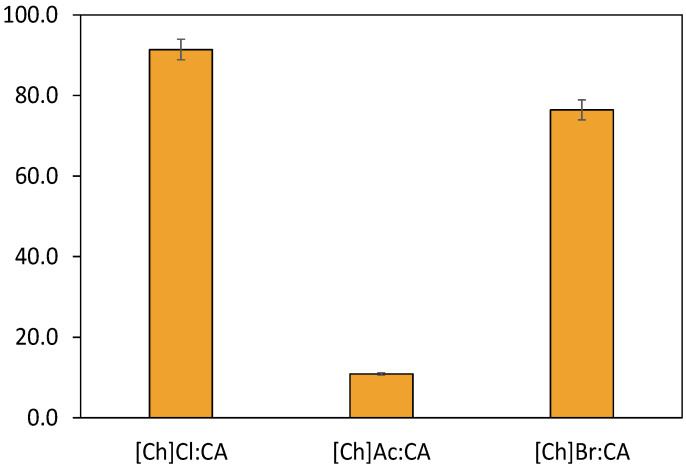
5-HMF yields attained for different cholinium-based HBA at 120 °C, 2 min of reaction and S/L ratio of 0.05.

In the next step of the DES screening, we tested different HBA anions to observe if it would be possible to boost even further the 5-HMF production yields. Figure 3 summarizes the 5-HMF yields obtained using DES media composed of CA and the HBAs [Ch]Cl, cholinium acetate ([Ch]Ac) and cholinium bromide ([Ch]Br) in a 1:1 molar ratio. The assays were performed in the following conditions: microwave heating for 2 min, 120 °C and solid/liquid (S/L) ratio of 0.05. As can be observed in Figure 2, the best 5-HMF yields were obtained with [Ch]Cl:CA, followed by [Ch]Br:CA and by [Ch]Ac:CA, with yields of 91.0%, 76.4% and 10.8%, respectively. These results are in agreement with the literature, in which the capability of the chloride anion to increase catalytic performance in the transformation of fructose into 5-HMF has already been discussed [23,37]. This anion has demonstrated the capability of increasing the system reactivity by ten-fold in aprotic solvents [37]. This increase is explained due to the lowering of the activation energy but also by the highly localized negative charge on the chloride anion, which in turn allows it to more readily stabilize protonated transition states of the oxocarbenium ion that forms in the acid-catalyzed reaction [37]. Taking these results into account, the DES [Ch]Cl:CA was selected to be used in the RSM approach to optimize the operational conditions of this process.

### 2.2. Reaction Optimization through Response Surface Methodology

With the goal of further optimizing the process and the different parameters associated, a response surface methodology (RSM) was applied. Time and temperature were selected for the RSM since they are evidenced in literature as determining parameters [5,35]. The third parameter selected was the use of a co-solvent to decrease the viscosity of the DES media namely which manipulation proved difficult in the initial assays. GVL was selected since it has been reported as being able to decrease the activation energy in these types of reactions [21] and promote 5-HMF production when used in the reaction media [38]. The impact of the addition of GVL in the reaction media was quite notorious, with the viscosity dropping from 495.68 (neat DES [Ch]Cl:Citric Acid (1:1)) to 240.08 mPa.s (media with the ideal amount of GVL (10%, see below)) at 70 °C and from 221.6 to 93.01 mPa.s at 90 °C.

**Figure 4:**
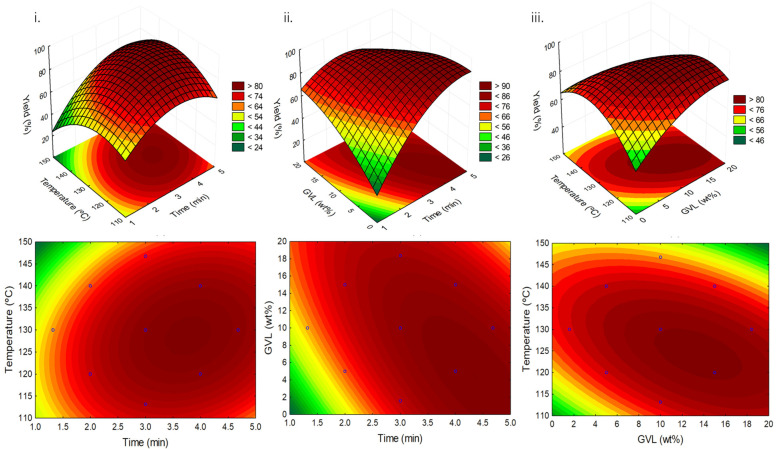
Response surface (top) and contour plots (bottom) of the 5-HMF yield using DES [Ch]Cl:CA (1:1) with the combined effects of: (**i**) temperature and time; (**ii**) GVL wt.% and time; and (**iii**) temperature and GVL wt.%.

Therefore, the temperature (°C), time (min) and GVL concentration (wt.%) were optimized by RSM. A 2^3^ (3 factors and 2 levels) factorial planning was carried out, being described in detail in Tables S1–S3 in the Supporting Information. This methodology allows the optimization of the relationship between the response (5-HMF yield) and the independent variables/conditions (temperature (°C), time (min) and GVL concentration (wt.%)). The influence of these three variables on 5-HMF yield is illustrated in Figure 4 and in the Pareto chart given in Figure S1 in the Supporting Information. Variance analysis (ANOVA) was used to estimate the statistical significance of the variables and their interactions. The experimental points used in the factorial planning, the model equation, the 5-HMF yield obtained experimentally and the respective calculated values, and the correlation coefficients obtained, as well as all the statistical analyses, are shown in Tables S1–S4.

As observed in Figure 4i,iii, temperature is a determining factor in the 5-HMF yield. This observation is also corroborated by the Pareto chart displayed in Figure S1 in the Supporting Information that reveals that temperature is the most relevant parameter. This parameter also presents a *p*-value below 0.05 which ascertains its significance within the model applied. It is thus clear that there is a positive effect in an increase in temperature up to 135 °C; however, at higher temperatures, the yield decreases. The observed decrease is related to the degradation of 5-HMF, which is a non-stable compound and is sensitive to the energy input into the reactional system [22]. These results are in agreement with previously reported studies dealing with the influence of temperature in HMF yield [6].

Time is the second most relevant parameter, according to the Pareto chart, and through the analysis of Figure 4i,ii. In Figure 4ii the highest 5-HMF yields are attained in a time window spanning from 3.5 to 5 min, while in Figure 4i the time interval narrows considerably when taking temperature into account. Consequently, higher 5-HMF yields are attained between 3 and 4 min of reaction time. Finally, GVL wt.% is the least relevant parameter as shown in the Pareto chart, being the same observed by analyzing Figure 4ii,iii. The results attained reveal that the addition of GVL has a similar effect from 10 wt.% up to almost 18 wt.%. Nonetheless, and once more, this range is narrowed when temperature is considered (Figure 4iii). It is also interesting to see that the use of GVL could be tailored to the other conditions. For example, the amount of GVL can be varied according to the reaction time, as displayed in Figure 4ii, with an inverse relationship. Since the temperature is fixed at 130 °C in this Figure, the low yields with smaller amounts of GVL could be related to 5-HMF degradation. On the other hand, at lower temperatures, an increase in the amount of GVL used could contribute to increase 5-HMF yields, as seen in Figure 4iii.

This behavior is interesting since it can make the process more tailorable, especially in the cases in which there are limitations to the energetic input. The observed results are also confirmed by the Pareto Chart in which the *p*-values are higher than 0.05 for both GVL and time. This is mostly due to the intrinsic relationship between parameters and the combinations that result in similar yields.

In this work, through the analysis of both the response surface methodology and respective Pareto chart, the best determined conditions are as follows: 130 °C, 4 min of reaction time and 10 wt.% of GVL using the DES [Ch]Cl:Citric Acid (1:1) in an S/L ratio of 0.05 with fructose. The model and therefore the result attained is validated by the calculated interception which presents a *p*-value below 0.05 (Table S4). These conditions were experimentally applied, resulting in a yield of 82.4 ± 2.6%, thus attesting the conducted optimization process (±3.1% deviation from the predicted value).

Despite the yield improvements expected based on previous studies, the yields obtained in the present study, when using GVL as co-solvent are lower than the yields obtained with neat DES (91.03%), nonetheless, there are advantages in using GVL, namely the decrease in solvent viscosity discussed above, as the use of GVL facilitates the handling of reaction mixtures. The addition of GVL will ultimately result in a decrease in the system reactivity due to the decrease in acidity and consequent yield decrease. Nevertheless, the yield attained is similar to what has been attained in literature featuring alternative solvents, namely ILs and DES [9,34]. However, the reactions times here achieved are much shorter due to the use of microwave irradiation (1 and 2 h vs. 4 min). Furthermore, the chosen DES can act both as a solvent and a catalyst, can improve reaction conditions using a bio-based solvent instead of common organic solvents (e.g., acetonitrile) [29] and does not need the addition of other catalysts such as metal halides (e.g., PtCl_4_, FeCl_3_, SnCl_4_) [5].

2.3. 5-HMF Recovering and DES Reuse

**Figure 5:**
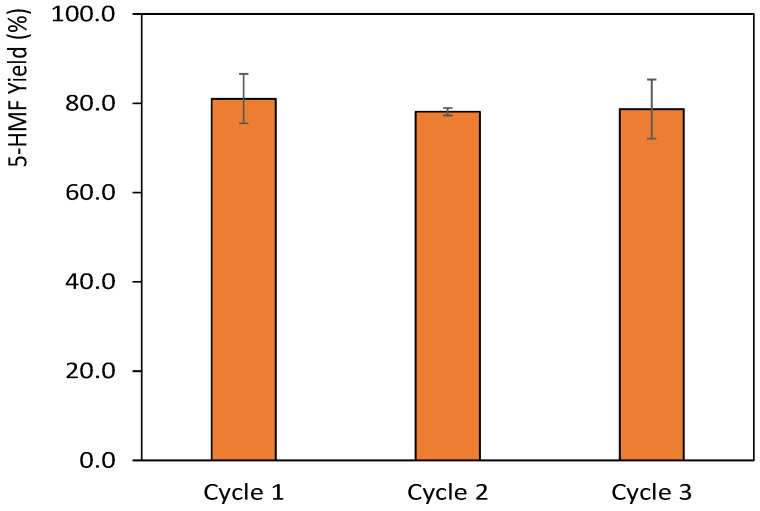
5-HMF yields attained through 3 cycles involving the solvent reuse of the system composed of [Ch]Cl:Citric Acid (1:1) with 10 wt.% of GVL. The microwave-assisted reactions were carried out at 130 °C with 4 min of reaction and S/L ratio of 0.05.

After the optimization of the 5-HMF production, it is crucial to focus on its recovery from the DES media to guarantee the development of a sustainable process. In this sense, the bio-based solvent 2-MeTHF was used to form a biphasic system with the DES-GVL media, thus allowing to extract 5-HMF to the organic phase. Through this method, it was possible to separate the DES from 2-MeTHF and GVL in a simple way. Finally, the use of fractional distillation under reduced pressure allowed the separation of 2-MeTHF from 5-HMF and GVL (boiling points 80 °C, 101 °C and 208 °C, respectively [39]. 5-HMF was recovered from the mixture by fractional reduced pressure distillation at 40 °C at 6 mbar. The temperature was kept as low as possible to prevent degradation of 5-HMF. The purity of the obtained compound was confirmed through ^1^H and ^13^C NMR and by comparison with standard 5-HMF, as provided in the Supporting Information–Figure S2. The solvent was recovered and reused, and this method was applied in three consecutive cycles of reaction. The results obtained using the recovered solvents are depicted in Figure 5. Through the application of 2-MeTHF as extracting phase, it was possible to recover 84.8 ± 1.2% of 5-HMF throughout the different cycles of reaction. The yields of 5-HMF do not decrease significantly with the DES reuse along cycles, with the yield in the last reaction being 78.7%. This reveals the feasibility of the designed process and the lack of the DES degradation, as also confirmed by the ^1^H and ^13^C NMR spectra presented in the Supporting Information–Figure S3.

## 3. Materials and Methods

### 3.1. Chemicals

Fructose (99% purity), tetramethylammonium chloride ([N_1111_]Cl, purity 98%) and DL-Lactic Acid (LA, 85% purity) were purchased from Acros Organics (Belgium). 5-Hydroxymethylfurfural (5-HMF, purity ≥ 99%), cholinium chloride ([Ch]Cl, ≥99% purity), levulinic acid (Lev, purity 98%), glycolic acid (GA, purity 99%) and γ-valerolactone (GVL, 98% purity) were supplied by Sigma Aldrich (USA). Citric acid mono hydrate (CA, purity ≥ 99.5%) and DL-malic acid (MA, purity 99.5%) were purchased from Panreac (Spain). Cholinium bromide ([Ch]Br, purity ≥ 98%) was purchased from TCI (Japan) and cholinium acetate ([Ch]Ac, purity 98%) was purchased from Iolitec (Germany).

### 3.2. DES Preparation

[Ch]Cl, [N_1111_]Cl, [Ch]Br and [Ch]Ac were used as hydrogen bond acceptors (HBAs) while citric, malic, levulinic, lactic and glycolic acids were used as hydrogen bond donors (HBDs). The HBAs and HBDs were weighted in glass vials in the desired molar ratio. The water content of both components was determined using a Metrohm 831 Karl Fisher coulometer and considered during weighting. The mixtures were then heated up to 70 °C and stirred for at least 1 h until a transparent liquid was formed. Mixtures were cooled down to room temperature and kept in sealed glass vials up to use.

### 3.3. Production of 5-HMF

An initial screening of different DES, namely [Ch]Cl:CA, [Ch]Cl:LA, [Ch]Cl:GA, [Ch]Cl:MA,[Ch]Cl:Lev, [N_1111_]Cl:CA, [N_1111_]Cl:LA, [N_1111_]Cl:GA and [N_1111_]Cl:MA, was carried out with microwave-assisted reactions. The reactions were carried out in a Monowave 300 microwave synthesis reactor from Anton Paar (Austria). The heating process took place for 2 min. Both the fructose and DES were weighted (S/L = 0.05) in appropriate sealed glass vials of 10 mL. Then, vials were placed in the microwave reactor at a fixed stirring of 600 rpm and removed at the end of the established reaction times (2 min) at 120 °C. After the assay was completed, the samples were left to cool down, then diluted appropriately with deionized water and filtered through a nylon Whatman filter of 0.45 µm pore into appropriate HPLC glass vials. All reactions were carried out in triplicate and the average yield and standard deviations presented. The best DES were then selected to test the HBA ([Ch]Cl vs. [N_1111_]Cl) and HBA anion influence ([Ch]Cl, [Ch]Br and [Ch]Ac).

### 3.4. 5-HMF Quantification

5-HMF quantification was carried out by HPLC-DAD (Shimadzu, model PROMINENCE). HPLC analyses were performed with an analytical C18 reverse-phase column (250 mm length × 4.60 mm diameter), Kinetex 5 μm C18 100 Å, from Phenomenex. The column oven and the autosampler operated at a controlled temperature of 35 °C and the mobile phase consisted of 20% of methanol and 80% of ultra-pure water. The separation was performed in isocratic mode, at a flow rate of 0.6 mL.min^−1^ and an injection volume of 10 μL. 5-HMF detection was carried out at 272 nm with a diode array detector (DAD). Calibration curves were established with pure 5-HMF aqueous standards. Each sample was analyzed at least in duplicate.

### 3.5. Reaction Conditions Optimization by Response Surface Methodology (RSM)

An RSM was applied to simultaneously analyze various parameters (reaction time (min), temperature (°C) and GVL concentration (wt.% relative to the total solvent weight)) with the goal of optimizing them and thus achieve the highest HMF yield. The description of the 2^3^ factorial planning used is provided in the Supporting Information-Equations S1 and S2. The obtained results were statistically analyzed with a confidence level of 95%. Student’s t-test was used to check the statistical significance of the adjusted data. The adequacy of the model was determined by evaluating the lack of fit, the regression coefficient (and the F-value obtained from the analysis of variance (ANOVA) that was generated. The Statsoft Statistica 10.0© software was used for all statistical analyses and representing the response surfaces and contour plots. From the interpretation of the RSM, it was then possible to narrow and interpret the effect of the different parameters in the HMF yield. The best conditions for HMF production were then selected by the analysis of the RSM results and compared. The best mixture composition was also compared in terms of viscosity with pure DES using an Anton Paar SVM 3000 viscosimeter. The measurements were acquired at 70 and 90 °C (the maximum temperature achievable by the equipment).

### 3.6. Recovery of the 5-HMF and DES Reuse

The bio-based solvent 2-MeTHF was utilized in a 2:1 weight ratio with the mixture to form a biphasic system and extract 5-HMF, that was then separated from the 2-MeTHF organic phase by fractional reduced pressure distillation. Since 2-Me-THF has a lower boiling point (80 °C) than 5-HMF (101 °C), it is first distilled followed by 5-HMF which has a lower boiling point than GVL (208 °C [39]). The distilled 5-HMF was recovered, weighed and the yield calculated. The recovered 5-HMF was then re-dissolved in water, diluted and filtered through a nylon Whatman filter of 0.45 µm pore into HPLC glass vials. The recovered DES-based solvent was used in at least three more cycles of 5-HMF production to address the effect of the solvent reuse on 5-HMF production yield. The solvent’s stability was followed through ^1^H and ^13^C NMR. The obtained 5-HMF was compared with a reference sample of furfural by ^1^H and ^13^C NMR. The ^1^H NMR and ^13^C NMR spectra were recorded using a Bruker Avance 300 at 300.13 MHz and 75.47 MHz, respectively, using deuterated water as solvent and trimethylsilyl propanoic acid (TMSP) as internal reference.

## 4. Conclusions

In this work, a simple, fast and efficient process for 5-HMF production from fructose was developed, namely under microwave-assisted reactions and using the DES [Ch]Cl:CA (1:1) that act as both catalyst and solvent. The process was optimized by varying several parameters, such as time, temperature, co-solvent concentration and HBA and HBD nature in the DES. The final optimal conditions achieved were [Ch]Cl:CA (1:1) at 130 °C, S/L ratio 0.05 and 4 min of reaction time and 10 wt.% of GVL. The use of the bio-based solvent GVL allowed for a decrease in viscosity of the solvent, thus facilitating the mass transfer phenomenon. Nonetheless, a more detailed study on the impact of the addition of GVL and particularly on the kinetic modeling of the reaction towards it detailed control will be the object of future studies. In these conditions, a maximum of furfural production yield of 82.4 ± 2.6% was attained.

The reuse of the DES was finally investigated and achieved through the use of bio-based solvent, 2-MeTHF, to extract 5-HMF from the DES medium. By fractional reduced pressure distillation, 84.8 ± 1.2% of 5-HMF could be recovered throughout the several cycles with high purity. Moreover, the DES was reused up to three times with minimal yield losses, showcasing the sustainability of the proposed process. This work is thus a steppingstone in understanding and optimizing processes for 5-HMF production, paving the way for their application to more complex substrates.

## Data Availability

Not applicable.

## Supplementary Materials

The following are available online at https://www.mdpi.com/article/10.3390/ijms23041959/s1.
