# Supplementary material for: Improved Production of 5-Hydroxymethylfurfural in Acidic Deep Eutectic Solvents Using Microwave-Assisted Reactions
Source: Int J Mol Sci. 2022 Feb 10;23(4):1959. doi: 10.3390/ijms23041959 (PMC8875992; doi:10.3390/ijms23041959)
Supplement: Supplementary file 1 [file ijms-23-01959-s001.zip › ijms-1538484-supplementary.pdf]

Supporting Information

Improved production of 5-hydroxymethylfurfural in acidic deep eutectic solvents using microwave-assisted reactions

Eduarda S. Morais[1], Mara G. Freire[1], Carmen S.R. Freire[1] and Armando J. D. Silvestre[1]*

[1]CICECO – Aveiro Institute of Materials, Chemistry Department, University of Aveiro, Portugal;

**Corresponding author: E-mail address: armsil@ua.pt; Tel: +351 234370701*

Process optimization – response surface methodology (RSM)

In a 2^k surface response methodology there are *k* factors that contribute to a different response, and the data are treated by a second order polynomial equation according to equation S1:

$$y = \beta_0 + \sum \beta_i X_i + \sum \beta_{ii} X_i^2 + \sum_{i<j} \beta_{ij} X_i X_j \qquad \text{(S1)}$$

where y is the response variable and β_0, β_i , β_{ii} and β_{ij} are the adjusted coefficients for the intercept, linear, quadratic and interaction terms, respectively, and X_i and X_j are independent variables. This model allows the drawing of surface response curves and through their analysis the optimal conditions can be determined (1). The 2^3 factorial planning has been defined by the central point (zero level), the factorial points (1 and −1, level one) and the axial points (level α) The axial points are encoded at a distance α from the central point, according to equation S2:

$$\alpha = (2^k)^{1/4} \qquad \text{(S2)}$$

Table S1. 2^3 factorial planning.

Experiment	χ_1	χ_2	χ_3
**1**	-1	-1	-1
**2**	1	-1	-1
**3**	-1	1	-1
**4**	1	1	-1
**5**	-1	-1	1
**6**	1	-1	1
**7**	-1	1	1
**8**	1	1	1
**9**	-1.68	0	0
**10**	1.68	0	0
**11**	0	-1.68	0
**12**	0	1.68	0
**13**	0	0	-1.68
**14**	0	0	1.68
**15**	0	0	0
**16**	0	0	0
**17**	0	0	0
**18**	0	0	0
**19**	0	0	0
**20**	0	0	0

χ_1 – time; χ_2– temperature; χ_3 – GVL concentration;

Table S2. Experimental data attained.

Experiment	χ_1	χ_2	χ_3	HMF yield (%)
**1**	2.0	120.0	5.00	75.19
**2**	4.0	120.0	5.00	85.72
**3**	2.0	140.0	5.00	67.94
**4**	4.0	140.0	5.00	81.78
**5**	2.0	120.0	15.00	93.16
**6**	4.0	120.0	15.00	80.80
**7**	2.0	140.0	15.00	68.82
**8**	4.0	140.0	15.00	69.18
**9**	1.3	130.0	10.00	44.18
**10**	4.7	130.0	10.00	94.65
**11**	3.0	113.2	10.00	66.84
**12**	3.0	146.8	10.00	73.44
**13**	3.0	130.0	1.60	70.80
**14**	3.0	130.0	18.40	80.67
**15**	3.0	130.0	10.00	86.81
**16**	3.0	130.0	10.00	90.21
**17**	3.0	130.0	10.00	84.10
**18**	3.0	130.0	10.00	90.84
**19**	3.0	130.0	10.00	83.57
**20**	3.0	130.0	10.00	81.33

χ_1 – time; χ_2 – temperature; χ_3 – GVL concentration;

Table S3. Coded levels of independents variables used in the first and second factorial planning.

Studied parameters	Symbol	Level				
		Axial	Factorial	Central	Factorial	Axial
		**-1.68**	**-1**	**0**	**1**	**1.68**
**Time (min)**	t	1.3	2.0	3.0	4.0	4.7
**Temperature (°C)**	T	113.20	120.0	130.0	140.0	146.8
**GVL (wt.%)**	GVL	1.6	5.0	10.0	15.0	18.4

Table S4. Regression coefficients of the predicted second-order polynomial model from factorial planning for the dependable variable of 5-HMF yield.

	Regression coefficients	Standard deviation	t-student (10)	P-value
**Interception**	85.91	4.216	20.373	< 0.05
**Time**	7.121	2.799	2.544	0.237
**Time^2**	-4.4522	2.728	-1.632	0.133
**Temperature**	-2.643	2.799	-0.944	< 0.05
**Temperature^2**	-4.194	2.728	-1.537	0.155
**GVL**	1.312	2.799	0.468	0.649
**GVL^2**	-2.213	2.728	-0.811	0.436
**Time × Temperature**	2.004	3.655	0.548	0.595
**Time × GVL**	-4.546	3.655	-1.243	0.241
**GVL × Temperature**	-3.0970	3.6552	-0.847	0.416

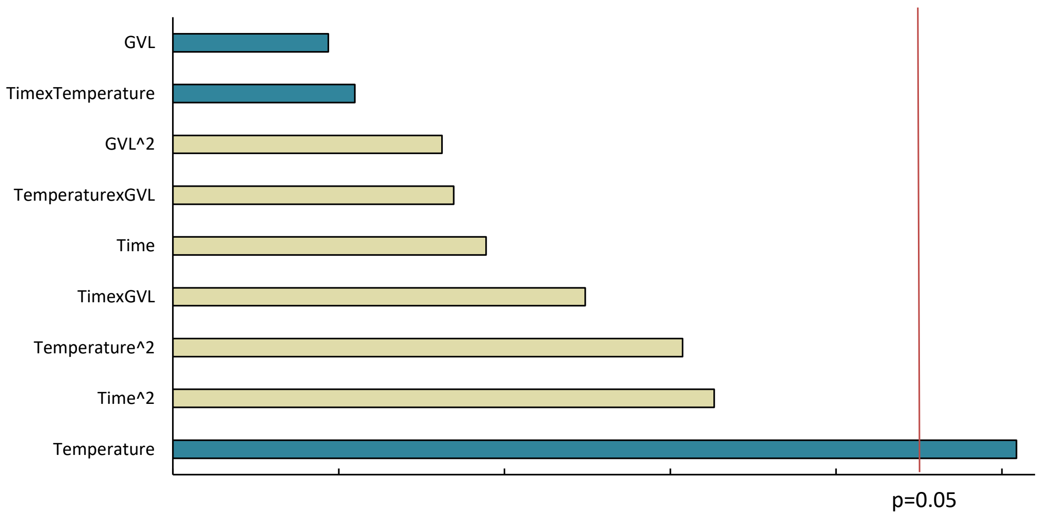

Figure S1. Pareto chart for the standardized main effects (positive ( ) and negative ( )) in the factorial planning for furfural yield optimization. Vertical line indicates the statistical significance of the effects.

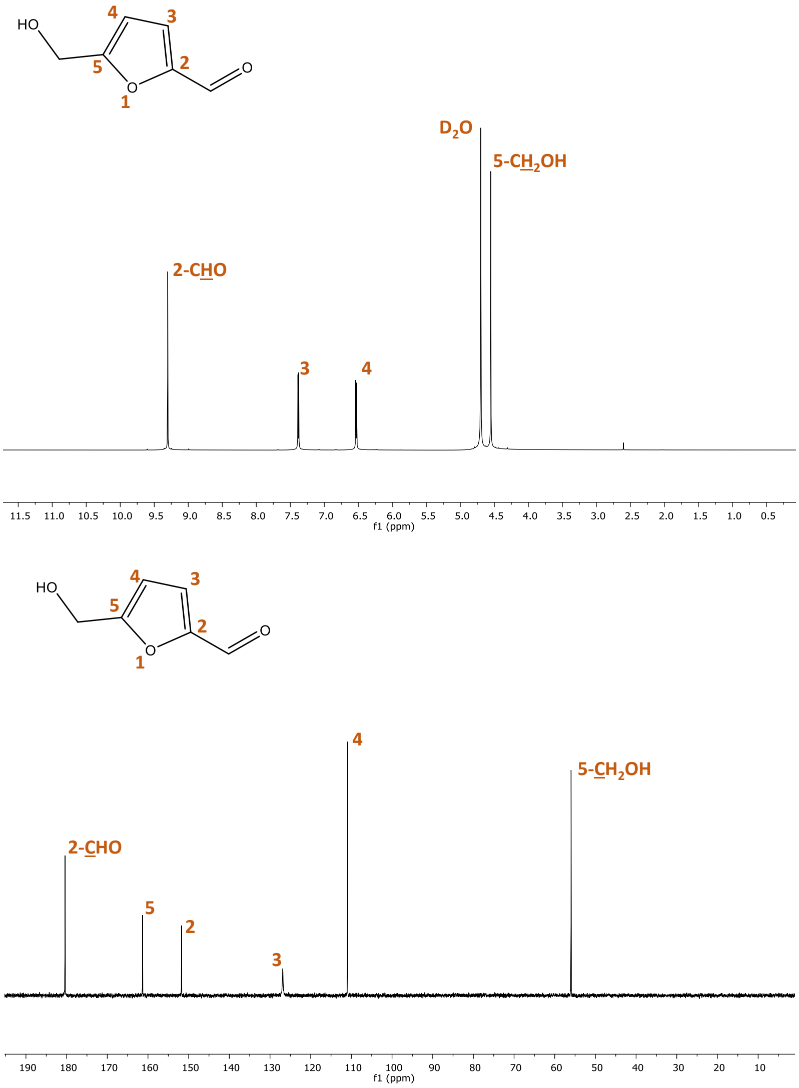

Figure S2. ^{1}H and ^{13}C NMR spectra of the recovered 5-HMF.

^{1}H NMR (D_2O, 300 MHz, [ppm]): δ 9.30 (d, 1H, 2-CH=O), 7.38 (d, 1H, H-3), 6.53 (dd, 1H, H-4), 4.55 (d, 2H, 5-CH_2OH). ^{13}C NMR (D_2O, 75.47 MHz, [ppm]): δ 180.38 (2-CH=O), 161.32 (C-5), 151.74 (C-2), 126.86 (C-3), 11.93 (C-4), 55.99 (5-CH_2OH).

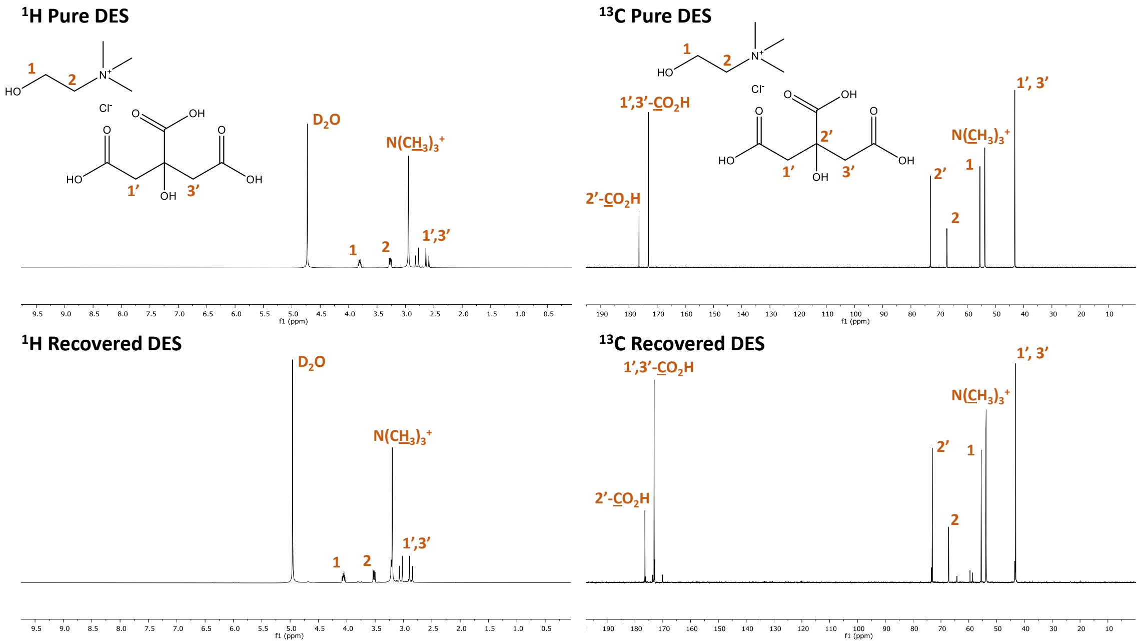

Figure S3. ^{1}H and ^{13}C NMR spectra of the pure and recovered [Ch]Cl:CA.
